# The Limits of De Novo DNA Motif Discovery

**DOI:** 10.1371/journal.pone.0047836

**Published:** 2012-11-07

**Authors:** David Simcha, Nathan D. Price, Donald Geman

**Affiliations:** 1 Department of Biomedical Engineering, Johns Hopkins University, Baltimore, Maryland, United States of America; 2 Institute for Systems Biology, Seattle, Washington, United States of America; 3 Department of Applied Mathematics and Statistics, Johns Hopkins University, Baltimore, Maryland, United States of America; National Institute of Environmental and Health Sciences, United States of America

## Abstract

A major challenge in molecular biology is reverse-engineering the cis-regulatory logic that plays a major role in the control of gene expression. This program includes searching through DNA sequences to identify “motifs” that serve as the binding sites for transcription factors or, more generally, are predictive of gene expression across cellular conditions. Several approaches have been proposed for *de novo* motif discovery–searching sequences without prior knowledge of binding sites or nucleotide patterns. However, unbiased validation is not straightforward. We consider two approaches to unbiased validation of discovered motifs: testing the statistical significance of a motif using a DNA “background” sequence model to represent the null hypothesis and measuring performance in predicting membership in gene clusters. We demonstrate that the background models typically used are “too null,” resulting in overly optimistic assessments of significance, and argue that performance in predicting TF binding or expression patterns from DNA motifs should be assessed by held-out data, as in predictive learning. Applying this criterion to common motif discovery methods resulted in universally poor performance, although there is a marked improvement when motifs are statistically significant against real background sequences. Moreover, on synthetic data where “ground truth” is known, discriminative performance of all algorithms is far below the theoretical upper bound, with pronounced “over-fitting” in training. A key conclusion from this work is that the failure of *de novo* discovery approaches to accurately identify motifs is basically due to statistical intractability resulting from the fixed size of co-regulated gene clusters, and thus such failures do not necessarily provide evidence that unfound motifs are not active biologically. Consequently, the use of prior knowledge to enhance motif discovery is not just advantageous but necessary. An implementation of the LR and ALR algorithms is available at http://code.google.com/p/likelihood-ratio-motifs/.

## Introduction

A major undertaking in computational biology is to reverse-engineer the cis-regulatory logic of the transcriptome that underlies much of gene regulation. Cis-regulatory logic controls transcription on the same DNA molecule as the regulatory logic. In contrast, trans-regulatory logic affects transcription of sequences that may be on a different DNA molecule. Examples of cis-regulatory logic include transcription factor (TF) binding sites, and signals that affect nucleosome positioning [1], DNA melting [2] and DNA methylation [3].

Properties of DNA sequences which are predictive of the “expression profile” of a gene or a related cis-regulatory property such as TF binding are referred to as “motifs”. Here an expression profile refers to the variation in the expression level of a gene across a variety of cellular conditions and the entire set of profiles may be quantized by assigning a gene membership in a cluster with other co-expressed genes; prediction then refers to identifying gene clusters from motifs. Canonically, motifs represent transcription factor binding sites (TFBS), but could be any predictive feature. Motifs may be single sequences, sets of sequences or probability distributions over sequences or over sequence features. The presence of a motif may be defined categorically (e.g., present or absent) or quantitatively (e.g., with reference to a probability distribution). The regulatory role of motifs may be investigated individually or interactively. Our focus is the discovery of individual motifs rather than learning combinatorial logic after initial motif discovery, as addressed for example by Beer et al. [4]. In addition, we only consider *de novo* motif discovery, meaning the discovery of motifs from an unbiased search of a set of sequences, rather than knowledge-based motif discovery, for instance using previously cataloged TFBS.

Unbiased validation of motifs discovered by computational methods is not straightforward because neither the locations nor nucleotide pattern of functional cis-regulatory elements are known completely. There are two statistical approaches to validation, one based on discrimination and the other based on hypothesis testing.

The discriminative validation method is to treat motif discovery as a discrete classification problem. This can be done if DNA segments near genes are partitioned into disjoint clusters. The goal is then to find motifs that can discriminate among, and thereby characterize, the clusters. There are two principal ways of defining clusters. One is to quantize their expression profiles by applying a standard clustering algorithm such as K-Means to gene expression profiles; in this case, the expression profile corresponds to the gene adjacent to the sequence. The other is to aggregate sequences based on the presence or absence of a transcription factor binding to the sequence, as determined by ChIP-chip or ChIP-seq experiments, forming nearly disjoint clusters. A motif associated with a given cluster is then validated by measuring its performance in distinguishing that cluster from all others, with appropriate adjustments for overlap in the ChIP case.

Common unbiased methods for measuring performance are cross-validation and hold-out validation. Both methods avoid the over-optimism inherent in measuring accuracy by resubstitution, which is the use of the same data for training and validation. This over-optimism, known as “overfitting”, is caused by the chance occurrence of patterns which are genuinely discrmininating in the training data but do not generalize to other data (e.g. held out samples) which are generated by the same mechanism or at least have the same statistical properties. We will show that these issues are pivotal in understanding the limits of *de novo* discovery. We use hold-out validation, which is both computationally simpler and generally more conservative than cross-validation. Data is partitioned into disjoint subsets and one is used for training and the other for validation. In our case, this means splitting each cluster of sequences (genes) into two groups, one used for motif discovery and the other for measuring classification performance. Notably, in the case of synthetic DNA sequence data with planted motifs, one can compute the Bayes error rate, or lowest error rate possible for a given classification problem, and related theoretical upper bounds on the performance of *de novo* methods.

Motifs can also be validated by statistical hypothesis testing. Properly done, this can improve interpretability by providing information on whether a motif is stronger than what one would expect to discover in a random set of DNA sequences. (The precise definitions of “stronger” and “random” vary depending on the method used, but the goal is conceptually the same.) Hypothesis testing is often neglected in the literature, especially when the motif model is probability-based (e.g., involves a position-weight matrix) rather than requiring an exact match to a pattern. When hypothesis testing is done, a generative null model is often used, which means defining a probability distribution over DNA sequences which represents “random” DNA not involved in any regulatory process. We argue (see Results) that the most common variants of these models are often “too null”. They fail to capture important properties of bulk, presumably non-regulatory, DNA. Especially in the human genome repetitive elements are prevalent. For example, repeats of the ALU element constitute approximately ten percent of the human genome [5]. Repetitive elements also have been shown to be highly conserved in some cases, with suggestive evidence of a regulatory role [6]. Therefore, masking such elements is questionable.

A second complicating factor in formulating a null hypothesis is the variation in low-order sequence properties such as dimer frequencies across clusters. Most methods do not consider these potentially discriminating signals; an exception is work that accounts for nucleosome positioning [7], which can be predicted by such low-order properties as the frequency of AA dimers. Assuming that low-order sequence properties are similar across clusters is one way that background models are “too null”; that is, they represent significantly less structure that exists in real biological sequences. Such properties appear (see Results) to be important predictors of expression profile and TF binding even by themselves. In addition to improving the biological relevance of hypothesis testing, using them as features might improve the performance of motif discovery algorithms.

These factors lead to motifs that are both statistically and biologically irrelevant being frequently declared significant. These kinds of discrepancies clearly reflect important differences between statistical significance (that varies relative to the chosen null hypothesis) and biological significance. Selecting better null distributions helps to align biological and statistical significance. In this article we argue that hypothesis testing based on discrimination is much more relevant than testing based on generative models. Here the null hypothesis is that a given motif is not overrepresented in a set of “foreground” sequences that share some property such as binding to a given TF or being upstream of a set of coregulated genes relative to “background” sequences, such as regulatory regions not adjacent to a coexpressed set of genes or not bound by the same TF as the foreground sequences. In this context repetitive elements are not problematic because, if a given element has no regulatory role, we expect it to occur in as large a fraction of the background sequences as foreground sequences.

In this paper, we explore the impact of background sequence models and variation of low-order sequence properties in validating existing motif discovery algorithms on both real and synthetic data, where we can compare the results obtained by *de novo* methods to the theoretical upper bound achieved when the correct motif is known. All methods are benchmarked using holdout validation, meaning evaluation of a classification rule using labeled data not used to train the classifier. To assess over-fitting, we also record the resubstitution (training set) error. We also introduce a new regression framework based on “mismatch” features (see Methods) designed to mitigate some of problems we have identified; adjusting for low-order sequence properties and an empirically-based null hypothesis leads to a small improvement in performance. However, in absolute terms we report uniformly low classification rates in predicting cluster membership as a proxy for expression profile or TF binding on all datasets. For synthetic data with know motifs, performance is far below the theoretical upper bounds given real-world sample sizes and sequence lengths.

Our results suggest discovering and validating motifs by attempting to predict expression patterns or TF binding computationally and without prior knowledge might be impossible due to the combination of several factors: the enormous size of the search space, the complexity of regulation, and the severely limited number of samples, namely the number of co-regulated genes identified with each expression profile cluster or the number of sequences bound by a given transcription factor. However, it must be emphasized this represents a *computational limitation*; since the theoretical upper bound is relatively high with real parameter settings, individual motifs could indeed be highly predictive were other biological signals incorporated into the discovery process.

### Motif Discovery Strategies

Except for recent work, the previous literature on motif discovery is well-reviewed [8]–[10]. In principle, motif discovery need not be treated as a classification problem. Instead, generative sequence models can be constructed to represent DNA not involved in regulation, and one can then search for patterns in real sequences that are over-represented relative to this model with respect to some expression or transcription factor binding property. This is the approach of several methods [11]–[14] which seek the most significant ungapped local alignments over a range of lengths within a set of sequences. A common generative model for random DNA is a Markov chain. Let 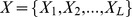 be a DNA sequence of length 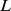, with 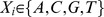. The 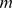
’th order Markov assumption is 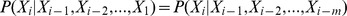. However, Markov models fail to account for large-scale variation in low-order sequence properties such as AT content, making hypothesis testing based on such models of dubious value (see Results). They also fail to account for repetitive and transposable elements, which constitute a large fraction of certain genomes.

Once a list of candidate sequences, say of length 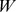, is determined by some motif discovery algorithm (for example, aligned portions of each sequence in a local alignment-based algorithm), the standard way of generalizing to a sequence distribution 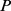 on 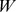-mers is a position-weight matrix or PWM. The matrix is 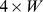: each row is a DNA nucleotide, each column a position, and the entry is the observed count in the set of sequences, possibly augmented with a pseudocount. Therefore each column represents the probability distribution of nucleotides at one of the 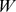 positions. Since the positions are assumed mutually independent, the log-likelihood of a 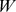-mer is computed by summing the log-probabilities of the nucleotides observed at each position, which can be compared with the log-likelihood under some background model. If the background model also assumes mutually independent components, the likelihood ratio test is the Naive Bayes classifier for a fixed 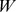-mer. When classifying an entire genomic sequence (e.g., the upstream region of a gene) according to membership in a gene cluster, high-likelihood 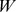-mers under the PWM serve as features.

Another approach [15]–[18] is to search for motifs defined by an exact match to a short sequence or regular expression. This may be done discriminatively [15], i.e. treating motif discovery explicitly as a classification problem, or using a background model in a fashion similar to the alignment method described earlier. In either case statistical hypothesis testing may be performed [15], [17], [18] since the relative simplicity of the model makes this tractable. However, in the discriminative approach to discovery, there is generally no systematic validation on data not used to train the classifier, e.g., no mention of estimating performance with holdout data or cross-validation.

Some attempts [19]–[21] classify sequences based on kernels. In particular, the *spectrum kernel* of depth 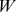 of a sequence 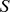 records the number of occurrences of each of the 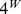 possible 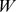-mers in 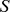; for instance, in [20], all 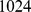 DNA 5-mers are considered as features. These methods are well-grounded in statistical learning and properly validated. However, it is difficult to perform hypothesis testing or to interpret the decision-making in biological terms, such as TF binding, due to the number and diversity of features.

A few important contributions fall outside these broad categories. For example, DME [22] formulates a discriminative model by enumerative perturbation of a PWM; ANN-Spec [23] learns a neural network; and DEME [24] learns a discriminative PWM model with a conjugate gradient algorithm. None of these approaches addresses statistical hypothesis testing or error estimation by holdout testing or cross-validation. Seeder [25] is a method that minimizes a suitable distance between a seed and a larger sequence and bears some resemblance to the LR algorithm (see Methods) introduced here. Whereas hypothesis testing and false discovery rates are explicitly addressed, there is no consideration of holdout-validation or cross-validation.

## Methods

### Datasets and Preprocessing

We consider three datasets: Yeast expression profile clusters from Beer et al. [4]; human gene expression data from the Connectivity Map project [26]; and ChIP-chip transcription factor binding data from Harbison et al. [27]. Here, an expression profile is the collection of mRNA concentrations for a set of transcripts (or a surrogate for this quantity, such as microarray hybridization intensity) under a predetermined set of conditions and a cluster of genes corresponds to a coarse quantization of profiles. The upstream regions of a cluster of coexpressed genes are assumed to be enriched for active binding sites (those that are accessible and actually bound *in vivo* under the relevant cellular conditions) for the transcription factor(s) responsible for the coexpression. Inactive TFBS, e.g. those not accessible to transcription factors due to chromatin structure or those that occur in the wrong context to modulate expression, are assumed to occur no more frequently in the upstream sequences of coexpressed genes than in randomly chosen upstream sequences. For the Beer data, 49 expression profile-based clusters were already specified based on the K-means algorithm [28]; we searched for motifs in the first 800 nucleotides upstream of the coding start site of each gene. For the Connectivity Map data, we generated 100 expression profile clusters using the K-means algorithm with Kendall’s Tau [29] as a distance metric, and examined the first 2000 nucleotides upstream of the most upstream coding start site of each gene for motifs. For the Harbison et al. data, only the rich media data (binding affinities of 175 TFs) were used and the set of sequences binding to a given TF was treated as a cluster. Genome annotations were obtained from the UCSC Genome Browser [30]. In all cases clusters of fewer than ten genes are excluded from the analysis due to excessively small sample size. Table 1 summarizes the key properties of each dataset.

**Table 1:** The properties and sizes of the datasets used.

Dataset	N Clusters	N Seqs	Clustering Method	Sequences
Beer et al.	49	48	K-means, Pearson Correlation	800 BP upstream of coding start
Harbison et al.	175	128	ChIP-Chip TF Binding	Binding seqs provided by Harbison et al.
Human CMap	100	100	K-Means, Kendall’s Tau	2000 BP upstream of coding start

N Seqs is the number of clusters that contained at least ten sequences.

Clusters with fewer than ten sequences were excluded from the analysis due to excessively small sample size.

### Classification Benchmark

Motif discovery algorithms can be validated by first grouping genes into disjoint clusters based on either similar expression profiles or common transcription factor binding and then attempting to assign cluster membership based on the existence of motifs. To quantify the regulatory predictive value of discovered motifs, for each cluster 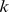 we estimated the accuracy of the motif learned using 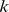 as the foreground cluster in discriminating between members and non-members of cluster 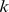. This was done using holdout validation, or validation on labeled data disjoint from the training data. Each cluster was partitioned into two disjoint and equally-sized subsets, one for training and one for validation. When discovering a motif in cluster 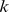, 200 sequences (100 training, 100 validation) randomly selected from the union of all clusters except 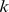 served as the “background” cluster. For the Harbison et al. data, since the clusters were not perfectly disjoint, sequences that appeared in the foreground cluster were excluded from the background cluster. For foreground clusters larger than 200 sequences, we randomly sampled 200 sequences to represent the cluster, for computational reasons. We used area under ROC curve (AUROC), a common performance metric in statistical learning, to measure the level of discrimination of the motif-based classifier for each cluster 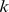.

All methods tested return a set of sequences of fixed length 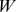 that represent samples from the motif discovered. (The ALR method also returns information about differences in the bulk distribution between foreground and background.) For all methods a PWM with a pseudocount of 1 was built from the set of sequences returned. This was used to produce the PWM score for each sequence 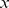. For simplicity, the PWM score was defined simply as the maximum likelihood of any subsequence of 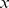 or its reverse complement, 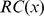. The PWM score 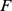 can be described as the maximum of the following two quantities:
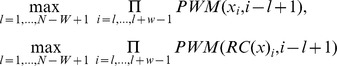



For all methods except ALR, the ROC curve and the AUROC value are generated by thresholding 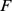. For the ALR method, 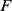 is combined with the bulk sequence features in a logistic regression model and the probability predicted by this model is thresholded to form the ROC curve. (The details of the ALR model are included later in this section.).

### Planted Motif Simulations

The objective is to measure the performance of *de novo* methods, as well as the theoretical upper bound, when the true motif is generated from a realistic PWM and the complexities of the background model are removed. We used *Saccharomyces cerevisiae* PWMs obtained from JASPAR CORE [31] with width 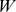 of at least eight nucleotides. These motifs were randomly planted in background DNA with sequence lengths and cluster sizes identical to the Beer et al. data, but generated from a zero-order Markov (or independent nucleotide) model with GC fraction equal to 0.4. For each cluster, a different motif was randomly chosen from among the available JASPAR motifs, and then inserted at a randomly chosen position in each member sequence by sampling independently from the distribution represented by each column of the PWM.

The theoretical upper bound on the AUROC performance of *de novo* methods was computed by assuming the true PWM was discovered and applying the classification benchmark described above. We used the same sets of foreground and background sequences for this benchmark as for the *de novo* benchmarks and reported the results on the sequences used for holdout in the *de novo* benchmarks so that the results would be directly comparable.

We also computed the accuracy of the Bayes rule for the full multiclass problem, i.e., for predicting which of the 49 clusters each sequence belonged to. We did this under the assumption of equal prior probabilities for all clusters. Since the cluster sizes vary significantly, using the true proportions would be overly optimistic. Since the positions of the motif were generated independently and every nucleotide of the background DNA was generated independently, the conditional independence assumption made by the naive Bayes classifier is in force, and hence the accuracy of naive Bayes is in fact the best possible (Bayes optimal).

Specifically, assume a sequence 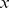 of length 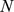 is scored for membership in cluster 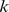. If 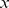 is a member of cluster 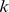 then a motif of length 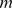 represented by 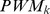 has been planted starting at a random position 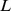 in 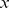. The rest of 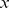 was generated independently with each nucleotide having probability 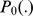. Let 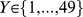 denote the true cluster. The probability that 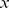 is a member of 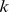 is proportional to the sum over all positions in 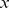 of the probability of observing 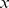 given that the motif represented by 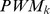 was planted at positions 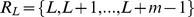:
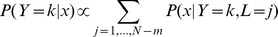


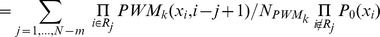



Here, 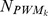 is a normalizing constant representing the number of sequences 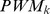 was built from, plus the pseudocount. This can be computed exactly for any cluster 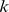 and sequence 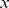 and the Bayes classifier is simply 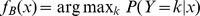, whose accuracy on the simulated dataset can also be easily computed.

### Logistic Regression

We introduce a new method for motif discovery based on logistic regression (the LR algorithm), which allows rigorous hypothesis testing, including multiple testing correction, but only utilizes real sequences, not generative background models.

Given two clusters 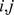, the goal is to find a PWM motif of some pre-specified width 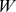 that discriminates members of 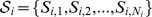 (the foreground cluster) from members of 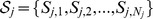 (the background cluster). (This method can be generalized to the case where the exact width of the motif is unknown by running it for multiple values of 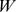 and choosing the most significant result.) Let 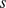 be some fixed sequence of length 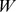 representing a candidate “core” motif and let 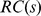 denote its reverse complement. The distance 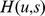 between 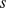 and any other sequence 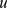 of length 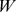, is taken to be the minimum of the Hamming distances from 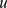 to 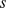 and from 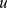 to 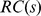. For any larger sequence 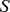 containing 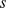, define
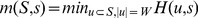
(1)which is minimum distance to 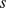 within 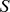. Consider the set of distances to the sequences in 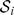:
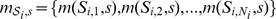
(2)and similarly for 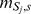. We refer to these as “mismatch” statistics. These are the features we regress upon.

Now consider a logistic regression model designed to discriminate between members of 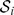 and 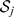 based on the mismatch features 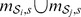. Let 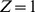 for sequences in 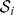 and 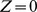 for sequences in 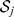. For each fixed sequence (candidate core motif) 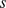, we regress 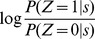 on the mismatch for 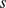. The parameters are learned by maximum likelihood. Let 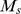 denote the model, which includes an intercept coefficient 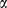 and a mismatch coefficient 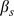, and let 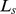 denote the log likelihood of the data under the model. We also learn a null model 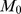 that includes only the intercept coefficient; let the likelihood of the data under this model be 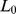.

Since 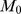 is nested in 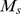 (the two models become equivalent if 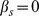), we use Wilks’ Theorem [32] to test 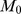 vs 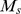 for each 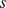 of width 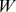. The test statistic is 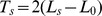 which is asymptotically 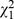-distributed under 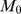 since constraining 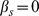 removes one degree of freedom. This procedure can be repeated either for every length 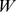 sequence or (for computational reasons) some subset thereof. In this paper every length 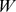 subsequence in 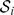 is used and the element of 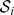 that the current 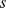 was obtained from is excluded from the hypothesis testing stage to avoid bias. This procedure results in a well-defined number of hypothesis tests, each assigned a p-value 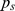. (In fact, a false discovery rate [33] can be computed for any set of discovered motifs.) We only keep the most significant motif 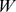-mer 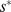, and learn a PWM from all length 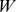 subsequences 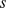 in any member of 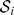 such that 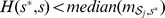. In other words, the idea is to create a PWM from all foreground subsequences that match 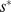 better than any subsequence in the majority of sequences in the background set as assessed by median Hamming distance to 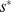.

Computing 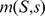 can be accelerated by preprocessing each 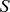 into a trie (also known as a prefix tree). The trie can be built in 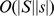 time but needs to be built only once for each 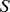 with the cost being amortized over multiple values of 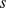. If 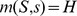, the worst-case time complexity of computing 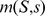 after the trie is built, for a four-letter DNA alphabet, is 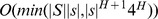. In practice this is extremely efficient because 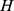 is usually small, and for large 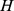 the worst case time complexity is equivalent to the naive algorithm of directly computing the Hamming distance between 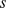 and every length 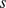 subsequence of 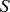.

The above model can be generalized to account for systematic variation in low-order sequence properties across clusters. We refer to this as the adjusted LR or ALR method since we attempt to find the most significant 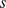 *conditioned* on these low-order properties. Start with a set of some pre-determined size 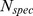 of features chosen from the feature space of low-order (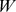 = 1 and 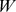 = 2) spectrum kernels. The set of features used is the 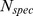 features that are individually most predictive, with a sequence and its reverse complement treated as identical. More precisely, define 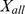 as the feature space of the union of spectrum kernels of 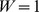 and 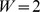, and let 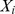 represent the 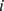
th feature. For each 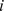 regress 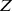 (as defined above) on only 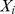 and an intercept term and choose the 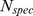 features that produce the largest likelihoods in such models. Biologically, these are intended to represent bulk properties of the relevant DNA sequence, such as nucleosome affinity, melting ability and flexibility. Such low-order properties are individually significantly correlated with cluster membership. (See Results).

Finally, we redefine the null model 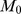 to include both the intercept coefficient and 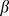 coefficients for 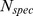 low-order spectrum kernel features. The model 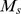 contains all of these features and additionally 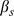 for some length 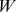 sequence 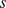. *The idea is to test whether anything additional is learned by adding information about 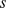 after accounting for low-order sequence properties.* 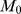 is still nested in 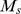 and Wilks’ Theorem can be used in the same way as above. A PWM is built from the highest scoring 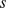 as described above, and a final decision rule is learned that combines the spectrum kernel features with the PWM score. This is done by creating a logistic regression model with an intercept coeffient, one 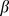 coefficient for each of the 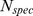 low-order features and one 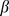 coefficient for the PWM score of the PWM built from 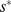.

### A/T Fraction Test

We performed a *Monte Carlo* test to assess whether Markov models are “too null” by testing whether A plus T fraction varies more across different contiguous genomic sequences than could be explained by sampling variance under a single global Markov model of non-coding DNA. Let 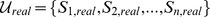 be a set of real DNA sequences used to train an 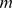
’th order Markov model, assumed to accurately capture the low-order properties of all sequences in 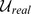.

Now, define 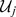 for 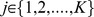 to be a set of synthetic sequences 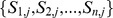 sampled from this Markov model, where 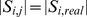. Define 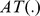 as the fraction of A plus T nucleotides in a given DNA sequence. For a sequence sampled from a Markov model with biologically realistic parameters, the A/T fraction will be approximately normally distributed. Let 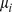 and 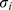 be the mean and standard deviation, respectively, of 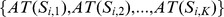. If a single Markov model provides an adequate description of the low-order properties of real non-coding sequences, 
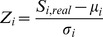
 should be distributed approximately 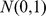.

## Results

### Even High-Order Markov Generative Models are Too Null

Monte Carlo simulations were performed to quantify the extent to which high-order Markov background models capture the structure of randomly selected sets of DNA sequences. MEME [11] was run approximately 15,000 times. We set the parameters to search for motifs of width 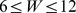 and to not search for multiple occurrences of a motif, but rather to only consider two possibilities: the existence of one motif or none. MEME was applied to random gene sets from the union of the Beer et al. [4] clusters. The size of each gene set was chosen as half the size of a randomly chosen cluster from [4]. (The other half of the data was used to verify that the motif discovered does not predict cluster membership on held-out data, or in other words that our “null” model is actually null.) This protocol allows for a null model where random sets of input sequences were used, but the individual sequences and the cluster sizes were real. For this analysis, the sequences are the upstream region of each gene from the coding start site to the next upstream transcript on the same chromosome.

**Figure 1:**
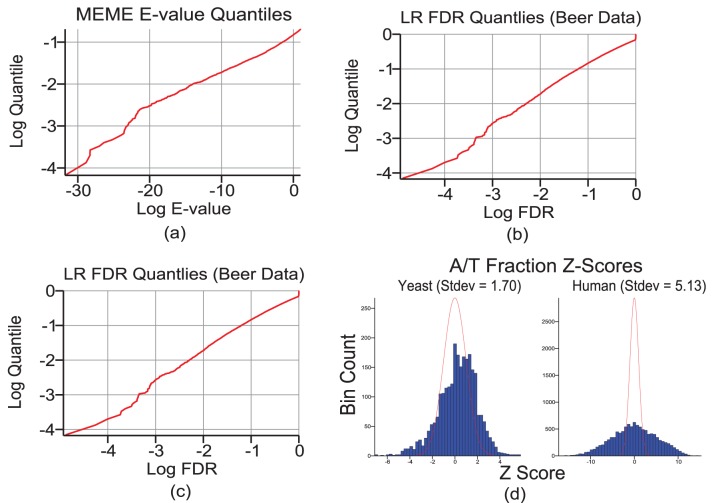
Generative models are too null. Panel (a): Quantile plot of Meme E-values for approximately 15,000 random runs, with E-values 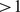 excluded. The X-axis represents the 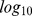 E-value as reported by MEME. The Y-axis represents the 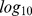 quantile. For example, under our null model E-values below 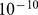 are reported with probability slightly more than 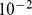. Panels (b) and (c): Quantile plots of LR false discovery rates, similar to the Meme E-value quantile plots, for the Beer et al. and Human Cmap datasets respectively. Panel (d): Z-score plots of A/T fraction of yeast and human intergenic sequences relative to the distribution expected under a 6th order Markov model, with the standard normal distribution (red) shown for reference.

The background model used was the 6′th order Markov model of yeast intergenic regions, which is included with MEME. MEME reports an E-value, which is the expected number of motifs at least as strong as the one observed under the null model, and which always exceeds the corresponding p-value. In this case the null (respectively, alternative) hypothesis is that no (resp., at least one) over-represented motif exists in the gene set. Under the null, p-values should be uniformly distributed over 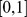. A similar test was performed using the LR algorithm with the same data set, as well as the 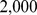 nucleotides upstream of the coding start site for genes in the Human Cmap dataset. Disjoint random gene sets were used for 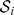 and 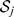. The FDR as computed by the LR algorithm also controls the family-wise error rate (the probability of making at least one Type I error) if all null hypotheses are true [33], as is the case in this *Monte Carlo* simulation. Therefore, an estimated false discovery rate 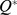 for the most significant motif should occur with probability no greater than 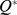. Under our null model of random sequence clusters, the E-values reported by MEME are anti-conservative even if interpreted as p-values (Figure 1a). Figures 1b and 1c demonstrate that false discovery rates produced by the LR algorithm are approximately accurate when all null hypotheses are true.

As described in Methods, a test for overdispersion of A/T fraction relative to the variance expected under a high-order Markov model was also performed. We used the first 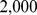 nucleotides upstream of the transcription start site for each gene in the human Connectivity Map [26] gene set and the full upstream intergenic regions of all yeast genes in the Beer et al. [4] dataset, ignoring regions with fewer than 100 nucleotides. A 6′th order Markov model was applied to all yeast sequences and another one for all human sequences. If all upstream sequences in each dataset were well-approximated by a single Markov model, the distribution of Z-scores would be expected to be approximately 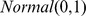. However, this is not the case (Figure 1d). There are at least two possible explanations: either even a 6′th order Markov model does not capture low-order properties of intergenic sequences, or such a model would not have homogeneous parameters across genomic regions.

Finally, low-order sequence properties differ significantly across clusters, as illustrated in Table 2, further supporting the hypothesis that commonly used generative background models fail to capture important structure. The fraction of each dimer in each sequence was regressed on cluster membership, with each dimer and its reverse complement being treated as identical. The value of 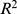 is the fraction of total variance in the frequency distribution of each dimer that is explained by cluster membership. The p-value tests the null hypothesis that the mean frequency of the dimer in each sequence is identical across clusters.

**Table 2:** The fraction of variance in dimer frequency across sequences explained by expression profile or transcription factor binding sequence set and associated F statistic P-value.

Dimer	Beer et al.	Harbison et al.	Human Cmap (Upstream)	Human Cmap (Introns)
AA/TT	0.076 (8.94e-21)	0.083 (4.47e-77)	0.110 (8.39e-208)	0.267 (0)
AC/GT	0.053 (7.38e-11)	0.057 (1.27e-32)	0.030 (6.09e-29)	0.068 (2.02e-102)
AG/CT	0.033 (0.000485)	0.056 (1.94e-30)	0.076 (2.24e-127)	0.210 (0)
AT	0.070 (6.34e-18)	0.148 (1.33e-222)	0.088 (1.84e-155)	0.231 (0)
CA/TG	0.037 (3.12e-05)	0.056 (5.35e-30)	0.078 (5.43e-131)	0.141 (2.01e-277)
CC/GG	0.115 (2.73e-40)	0.156 (7.28e-245)	0.101 (1.28e-186)	0.228 (0)
CG	0.093 (4.85e-29)	0.158 (2.15e-249)	0.081 (1.85e-139)	0.095 (4.04e-166)
GA/TC	0.047 (1.44e-08)	0.051 (1.25e-22)	0.041 (2.92e-49)	0.164 (0)
GC	0.078 (1.36e-21)	0.149 (1.56e-227)	0.081 (3.88e-139)	0.207 (0)
TA	0.051 (5.05e-10)	0.131 (5.53e-183)	0.098 (1.37e-180)	0.265 (0)

For the Human Cmap data, this was assessed both for the 2,000 nucleotides upstream of the coding start site and for the intron sequences.

The overall conclusion of these experiments is that common generative approaches may produce overly optimistic conclusions about the significance of motifs discovered because the null models are “too null”, or assume that bulk non-coding DNA is more random than it really is.

### Classification Rates of all Tested Methods are Poor

We evaluated the performance of five motif discovery algorithms in predicting gene cluster membership on three real datasets and one synthetic dataset. The methods are MEME [11], 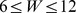, zero or one motif instance per sequence model; AlignAce [12], numcols = 12; DEME [24], 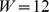; and the LR and ALR algorithms, with 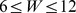 and 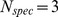 for ALR. Recall that the ALR algorithm accounts for the discriminating power of bulk DNA features, whereas the LR algorithm ignores low-order features and hence can be compared directly with the other three methods. The datasets are Human Cmap, yeast Beer et al., and yeast Harbison et al., and the simulated yeast dataset mentioned previously. In all cases both the forward and reverse complement strand were analyzed. For MEME, the background model was second-order Markov and estimated from the background sequences. For AlignAce, the background GC content was set to that the background sequences. Whenever a method output a list of sequences, these were converted to a PWM by calculating the empirical distribution over the four nucleotides at each position, except a unit pseudocount was incorporated to avoid zero probabilities. Table 3 displays the estimated mean holdout AUROC values across clusters for all datasets and algorithms. Table 4 displays the resubstitution AUROC.

**Table 3:** The mean AUROC of all algorithms on all datasets using independent holdout data.

Mean AUROC (Holdout)				
	Beer et al.	Harbison et al.	HumanCMap	Synthetic
LR	0.591	0.600	0.530	0.677
ALR	0.620	0.629	0.569	0.683
MEME	0.598	0.536	0.521	0.718
AlignAce	0.561	0.524	0.524	0.660
DEME	0.613	0.557	0.541	0.677

This validation is unbiased.

**Table 4:** The mean AUROC of all algorithms on all datasets based on training and testing on the same data.

Mean AUROC (Resubstitution)				
	Beer et al.	Harbison et al.	HumanCMap	Synthetic
LR	0.776	0.771	0.731	0.814
ALR	0.836	0.857	0.799	0.858
MEME	0.753	0.784	0.637	0.809
AlignAce	0.657	0.693	0.584	0.831
DEME	0.835	0.848	0.799	0.894

The optimistic bias reveals massive overfitting.

There is a wide range of classification rates from method to method and from dataset to dataset. The best performance in all real datasets is obtained by the ALR algorithm. This may be due to the regression framework, which accommodates hypothesis testing without a synthetic background model and learns bulk sequence properties in addition to PWMs. DEME, which also takes an explicitly discriminative approach to motif discovery, also fares relatively well compared with AlignAce and MEME, which use artificial background null models. However, the most striking trend is that all mean AUROCs are disappointing, reaching at most around 0.62 on real data and 0.72 on synthetic data.

To determine the theoretical upper bound classification accuracy we computed the mean AUROC on the synthetic dataset as in Table 3 but used the PWM motifs that we planted instead of discovered PWMs as classifiers. The mean AUROC here was 0.865. Furthermore, the multi-class Bayes accuracy for determining the correct cluster membership for each sequence from all 49 available clusters was 0.344. These results demonstrate the substantial discriminative ability of single motifs in this context and the theoretical possibility of achieving much higher one-versus-all discriminative power than any method achieved on any dataset.

### Proper Hypothesis Testing Predicts Generalization

The purpose of hypothesis testing is to determine which motifs may be biologically relevant signals and which are more likely statistical noise. We compared the mean AUROC for significant (FDR
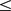
0.05) vs. non-significant (FDR
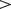
0.05) motifs using our LR and ALR methods and all datasets. Table 5 shows that statistical significance does predict generalization of a motif’s discriminative ability from the training set to the validation set. Note that ALR retains substantial discriminative ability in the absence of a significant motif because the bulk features also contribute to discrimination. LR, on the other hand, performs barely better than the 0.5 AUROC expected under random guessing.

**Table 5:** The mean holdout AUROC of the LR and ALR algorithms for motifs for non-significant (FDR
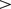
0.05) and significant (FDR
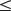
0.05) motifs respectively.

Mean AUROC (Non-Significant/Significant)				
	Beer et al.	Harbison et al.	HumanCMap	Synthetic
LR	0.531/0.722	0.562/0.656	0.510/0.569	0.536/0.796
ALR	0.571/0.727	0.580/0.697	0.562/0.587	0.521/0.790

## Discussion

This study has identified the following key findings: First, we have shown that even high-order generative models of random DNA are “too null” resulting in overly optimistic estimates of motif discovery in DNA sequences. Motif discovery methods should therefore be evaluated in a classification framework using only real DNA sequences. Second, we have shown that rigorous hypothesis testing can still be incorporated by providing a well-defined null hypothesis, namely that a motif is over-represented in a well-defined foreground cluster relative to a well-defined background cluster, where again both consist of real sequences. Given that this approach is discriminative, standard methods of validating a classifier (namely holdout or cross-validation) can and should be used. Applying this stringent benchmark, all methods perform poorly regardless of whether sequences are clustered by ChIP-chip TF binding or by mRNA expression. Explicitly discriminative methods and those that account for differences in bulk DNA properties have marginally improved discriminative ability. Thus, there is clearly much need for improvement in computational discovery of motifs. Given the statistical difficulties, it is clear that biological knowledge and the integration of high-throughput and heterogeneous data will not only be useful, but essential to ultimately achieving high accuracy. For example, accounting for chromatin modification [34] might aid in distinguishing between TFBS that are accessible and therefore potentially bound and those that are inaccessible to the relevant TFs under a given cellular condition.

The relatively high theoretical upper performance bound on the synthetic data suggests that the poor performance of *de novo* methods cannot be attributed primarily to low predictive value of individual motifs. Thus, the relatively poor performance on real data of methods that focus on finding individual motifs may not be, of itself, strong evidence that individual motifs are not biologically prevalent and meaningful in vivo. The gap between this upper bound and the performance of *de novo* discovery algorithms on the synthetic dataset is also much larger than the performance gap between *de novo* discovery on the real Beer et al. vs. synthetic datasets. The synthetic dataset has sequence lengths and cluster sizes identical to the Beer et al. data but conforms to our simplified biological model that exactly one motif exists in each cluster and that the motif is reasonably long and can be statistically modeled accurately by a PWM. These results suggest that statistical tractability is a more severe problem than deficiencies in the biological models used by discovery algorithms, such as ignoring combinatorial regulation or using PWMs instead of more complex models of motifs. Using more complex, biologically realistic models such as attempting to simultaneously discover motifs involved in combinatorial regulation or removing the assumption of conditional independence between positions that the PWM model implies would likely exacerbate these statistical issues. This is especially true since the sample size available for discovery is limited by the number of occurrences of a given cis-regulatory element in the genome. This limit can be effectively increased by using phylogenetic methods such as PhyloGibbs [35]. However, this assumes that cis-regulatory elements are mostly conserved across species and only increases sample size incrementally.

It might be argued that clusters based on expression of downstream genes or ChIP-chip TF binding are noisy representations of the set of sequences regulated by a given cis-regulatory mechanism, e.g. the binding of a specific TF. For example, TF binding has been shown to be context specific with regard to developmental stage [36] and ChIP data does not always accurately predict transcription factor binding events catalogued in the literature [37]. However, poor performance is observed regardless of whether clusters are defined by ChIP-chip transcription factor binding or expression clustering. Furthermore, results on the synthetic data, which uses planted motifs and thus guarantees that every member of a given cluster contains an example of the same TFBS, are only incrementally stronger than those on the Beer et al. data. Taken together, our results suggest that inaccuracies in clustering caused by imperfections in expression or ChIP-chip data cannot fully explain the poor performance. Furthermore, we argue that imperfect clusters represent a more realistic use case for *de novo* discovery methods than the ideal case where each cluster perfectly represents the set of sequences regulated by a given cis-regulatory mechanism, making our discriminative benchmark highly relevant.

The strong resubstitution performance (i.e. when the model is trained and tested on the same data), especially on the synthetic data, suggests that statistical tractability is also a more severe problem than optimization of the objective functions of the discovery algorithms. Such discrepancies between resubstitution error and error in a test set are clear indicators of overfitting issues.We have established an upper performance bound of approximately 0.865 on the synthetic dataset. All algorithms achieve an average resubstitution AUROC of at least 0.8 on this dataset, even though all use heuristics to optimize their objective functions. In other words, all algorithms on average find solutions with discriminative power comparable to the correct solution on the training data even though these often don’t generalize – as seen by greatly reduced AUROC in holdout validation.

The difficulty of developing adequate generative models of background DNA sequences may be partly due to the variation in low-order sequence properties across expression and transcription factor binding profiles. This phenomenon has previously been observed specifically with respect to GC content in human promoter regions [38]. Our results suggest that the phenomenon of low-order, large scale sequence properties being correlated with expression and TF binding is more broadly applicable, both to yeast and to the frequencies of a variety of dimers. They also suggest that the statistical significance of longer (e.g. 12-mer) motifs may sometimes result from of variation in the low-order properties of sequences across expression or TF binding profiles, and these would not be significant *given* the low order properties of the sequences in which they were found. Such motifs are not likely to be biologically meaningful.

Nucleosome positioning can be predicted by low-order sequence properties such as 1- through 4-mer frequences [1]. Nucleosomes appear to be depleted in active regulatory regions [39]. This observation and a study in the PH05 promoter [40] suggest that high nucleosome occupancy may interfere with transcription factor binding in at least some cases. If the main link between low-order sequence properties and gene expression were that low-order sequence properties affect nucleosome occupancy, which affects TF binding and in turn affects expression, then low-order sequence properties in small, specific regions would be expected to predict expression and TF binding, but not low-order properties across large (several hundred nucleotides or more) regions. Similarly, such properties would not be expected to be frequently predictive in regions such as introns, where TFBS occur less frequently than near the transcription start site (TSS).

### Conclusion

*De novo* DNA motif discovery remains a challenging problem with computational methods. The fact that common generative models, or explicit models of the probability distribution of “random” DNA, cannot represent “random” DNA with reasonable accuracy motivates a discriminative approach where real background sequences are used. However, applying traditional validation protocols for classification algorithms reveals universally disappointing rates in predicting expression profile or TF binding from sequence. The largest obstacle may be over-fitting, which will be difficult to overcome since the samples size is effectively the number of genes in strongly co-regulated clusters or bound by a given TF, and thus cannot be expanded arbitrarily to provide the necessary statistical power.
